# Stigma Levels Toward Psychiatric Patients Among Medical Students—A Worldwide Online Survey Across 65 Countries

**DOI:** 10.3389/fpsyt.2021.798909

**Published:** 2021-12-13

**Authors:** Mateusz Babicki, Monika Małecka, Krzysztof Kowalski, Bogna Bogudzińska, Patryk Piotrowski

**Affiliations:** ^1^Department of Family Medicine, Wroclaw Medical University, Wrocław, Poland; ^2^Department and Clinic of Psychiatry, Wroclaw Medical University, Wrocław, Poland; ^3^Students' Scientific Group at the Faculty of Psychiatry, Wroclaw Medical University, Wrocław, Poland; ^4^Section of Epidemiology and Social Psychiatry, Department and Clinic of Psychiatry, Wroclaw Medical University, Wrocław, Poland; ^5^Division of Consultation Psychiatry and Neuroscience, Wroclaw Medical University, Wrocław, Poland

**Keywords:** medical students, mental disorder, stigma attitude, human developing index (HDI) resource curse, mental health

## Abstract

**Background:** According to epidemiological data, over 450 million people worldwide suffer from mental disorders, presenting one of the major challenges of modern medicine. In their everyday lives, patients, in addition to fighting the disease itself, often struggle with stigmatization. This phenomenon negatively affects both the diagnostic and therapeutic processes, as well as the patients' everyday functioning. This study aimed to assess stigma attitudes toward psychiatry and psychiatric patients among undergraduate medical students.

**Methods:** This study used a Computer-Assisted Web Interview (CAWI), which included the standardized items from the Mental Illness: Clinicians' Attitudes (MICA-2) scale to evaluate stigma. The study was disseminated via the internet to students from medical universities from 65 countries worldwide. Participation was voluntary and anonymous. The study involved 1,216 students from these 65 countries. Most of the sample were women, and most were medical faculty students and students living in cities with more than 500,000 residents. Taking into consideration Gross Domestic Product (GDP) per capita and Human Development Index (HDI) variables, it can be seen that there was a prevalence of medical students from highly developed countries.

**Results:** For the whole sample, the mean MICA-2 score was 40.5 points. Women and medical and nursing students showed more positive attitudes toward psychiatric patients. Students from countries with the highest economic development levels also achieved statistically lower MICA-2 scores. Lower score means a more positive attitude.

**Conclusion:** Stigma toward both psychiatry and psychiatric patients is common among undergraduate medical students. Female students and respondents with a history of mental disorders in countries with high HDI and GDP per capita indices show more favorable attitudes than other medical students. There is a need to further our understanding of the problem of stigmatization, both among the general population and among medical personnel, and to implement and maintain appropriate measures to reduce stigma toward psychiatry.

## Introduction

According to epidemiological data, over 450 million people worldwide experience mental disorders, making these disorders one of the greatest challenges of contemporary medicine (1, 2). Furthermore, the most recent research is not optimistic and suggests that the number of psychiatric patients, especially those over 65, will continue to expand (3). According to recent projections, 6.5 million individuals will need psychological or psychiatric assistance in Poland in the coming years (1). Epidemiological studies on the European population estimate that around 27% of the continent's occupants will need psychiatric consultation throughout their lifetime (1). This will contribute to a substantial burden on the currently inefficient psychiatric care system (4).

Apart from the problems mentioned above, there are also factors conditioned by environmental and social aspects. In their everyday lives, patients, aside from dealing with their diseases, additionally fight stigmatization (5). Because stigmatization is a complex process, it is not feasible to specify its causes. However, the possible variables that might influence its prevalence are a lack of proper understanding about the nature of mental diseases and an increased proneness to succumb to stereotypes about psychiatric patients (6).

Another aspect behind stigmatization may be discomfort and fear during contact with patients and poor knowledge of how to behave appropriately in their presence (7).

In the last decade, much interest has been devoted to treating the enacted and perceived stigma toward individuals with mental illness as an indispensable component of the diagnostic and therapeutic process (8, 9). Typically, individuals struggling with psychiatric conditions are considered to be worse and less intelligent, and are perceived differently to others (10). According to general opinion, people with mental disorders are more disapproved of than those with physical health problems. They are presumed to be responsible for their condition and are often falsely accused of committing fierce criminal offenses (11).

A crucial aspect of discrimination in the economic domain shows up in an increased reluctance to employ people who are making use of psychiatric care services, which may decrease these individuals' socioeconomic status and, what is even worse, postpone the initiation of suitable therapy or perhaps even cause treatment of diseases for patients who respond well to treatment to be ceased (9). All this results in overt discrimination and contributes to the limitation of the patient's ability to meet suitable social roles, which are inevitably connected to the efficiency of mental health well-being rehabilitation.

It is also worth mentioning the self-stigma process that influences some psychiatric patients, especially those who share common views concerning their discrimination. Self-stigma causes a reduction in self-confidence, reduced self-esteem, social isolation, and is usually used as a psychosocial mechanism to protect independent functioning (6, 11). Previous reports clearly show an association between self-stigma and the severity of clinical signs and symptoms, along with limited participation during treatment (10).

Stigmatization is common both among the general population and among medical care employees (12). It has been revealed that medical students who will be future healthcare professionals likewise may contribute to the spread of stigmatizing stereotypes about patients dealing with mental illness. Among the stigmatizing stereotypes are beliefs about patients' aggression and unpredictability, which strengthens the social exclusion of these patients. Furthermore, medical students tend to evaluate the job of psychiatrist as stressful, overwhelming and emotionally exhausting (10, 12).

Nonetheless, this approach toward the mentally ill may change to some extent as education at medical school progresses. While it was confirmed that the penultimate year students present stigmatizing attitudes toward patients who have schizophrenia and also depression (12, 13), for that very reason, numerous research studies suggest that introducing holistic education and learning in medical universities and increasing straight doctor-patient contact may decrease manifestations of stigma attitudes amongst clinical personnel (13, 14).

Regardless of the increasing demand for mental health professionals, low recruitment for this specialization has been observed worldwide (15). According to the World Health Organization (WHO), there ought to be one psychiatrist for every 10,000 citizens in each country. The USA, most European countries, Australia, Japan, and New Zealand already satisfy this condition. Nevertheless, there are nations in Africa and Asia where this percentage is just 0.2 per 10,000 citizens, and there are countries with no psychiatric services at all (16).

It has been shown that medical students consider psychiatry to be the least respected expertise in terms of further professional development (4, 16). Therefore, there is an urgent need to implement techniques aimed at getting medical students interested in psychiatry, specifically to meet the needs of the healthcare system (3, 4).

It has been shown that the stigmatization process exerts a significant impact on the functioning of psychiatric patients, and that most of the variables affecting its occurrence arise from inadequate social awareness induced by a lack of psychoeducation in the society. It is, therefore, crucial to analyse the entire phenomenon comprehensively and to draw conclusions that will enable the implementation of appropriate measures to fight labeling, discrimination and depreciation driven by stereotypes, as well as to dealing with mentally ill individuals in a different way than the rest of society (6).

This study aimed to assess the extent of stigmatization beliefs in medical university students toward psychiatry research and psychiatric patients. Secondary goals were to examine how the socio-demographics of the student sample affected stigmatization, and how completing a training course in psychiatry could change these stigmatizing attitudes. Based on previous reports indicating the significant prevalence of the phenomenon of stigmatization both in the general population and among medical students, especially among men, the following research hypotheses were formulated (10, 16): (1) Stigmatization is a common attitude amongst medical students. (2) Female students show more favorable attitudes toward psychiatric patients than do male students. (3) Students from countries with a high Human Development Index (HDI) and high Gross Domestic Product (GDP) per capita reveal a greater extent of stigmatizing attitudes.

## Materials and Methods

### Study Participants and Settings

A Computer-Assisted Web Interview (CAWI) survey was addressed to medical students regardless of their faculty (medicine, dentistry, nursing, obstetrics, medical emergency) and the place and stage of their education. A survey designed by the authors included sociodemographic information of participants including age, sex, country of origin, information on medical schools, study course, year of education and whether or not they had completed a course in psychiatry. The individual and/or student's family history of mental disease was additionally analyzed.

The research was performed according to the guidelines of the Declaration of Helsinki and approved by the Bioethics Committee of the Wroclaw Medical University, Poland (Approval No: KB-178/ 2021).

### Computer-Assisted Web Interview (CAWI)

The CAWI study was based upon a questionnaire designed for this study and disseminated through the internet using social media. Participation in the research study was voluntary and anonymous, and the participants were not paid to finish the questionnaire. Before participating in the survey, respondents were presented with the most essential aspects of the study, including the methodology, purpose of the study, and approximate duration of the survey. After becoming knowledgeable about the study, respondents gave their informed consent to participate. In addition, all participants were informed of their ability to resign from the study at any phase without providing any reason.

### Mental Illness: Clinicians' Attitudes (MICA-2)

The following part of the study involves the standardized MICA-2 psychometric measure of stigma attitudes to assess the attitudes of students at medical schools toward both psychiatry as a field of study and patients with mental health disorders. The MICA-2 is based on a 6-point Likert scale. Questions 3, 9, 10, 11, 12, and 16 are scored as follows: “I Strongly Agree” = 1 point; “I Agree” = 2 points; “I Rather Agree” = 3 points; “I Rather Disagree” = 4 points; “I Disagree” = 5 points; “I Strongly Disagree” = 6 points. The remaining items are scored oppositely. The overall scores acquired by the participants ranged from 16 to 96 and indicated increasing degrees of stigmatization related to attitudes and beliefs expressed by the respondent. The MICA-2 scale shows high internal consistency and also reliability (17–19).

### Human Development Index (HDI) and Gross Domestic Product (GDP)

In order to examine the effects of socio-demographics, participants were split according to HDI and GDP groups based on data from the World Bank and the United Nations Development Program (20, 21). Groups were identified with very high, high, medium and low HDI indices, and were categorized by high, upper-middle, lower-middle, and low GDP income per capita.

### Statistical Analysis

Statistical analysis was performed using the STATISTICA 13.0 software (StatSoft, Hamburg, Germany). The variables were quantitative, qualitative, and dichotomous. Basic descriptive statistics were used to analyse quantitative variables. In order to examine the normality of the distribution, the Kolmogorov-Smirnov, Lilliefors, and Shapiro-Wilk tests were used with a statistical significance of 0.05. When the distribution was non-normal, the Mann-Whitney *U*, or Kruskal-Wallis tests were used. The analysis of covariance (ANCOVA) was utilized to examine the influence of confounders while assessing the group differences across nations broken down by GDP per capita and HDI, such as age, gender, medicine discipline, course completion in psychiatry, and also history of mental disorders. At each phase of the study, results were considered statistically significant at 0.05.

## Results

### Characteristics of the Study Group

The study included 1,216 respondents from 65 countries, of which 860 (70.1%) were medical students and 904 (74.3%) were female. The percentage of students who had not yet finished a training course in psychiatry was 54.3%. For GDP and HDI values, the most numerous groups were represented by countries with high-GDP per capita, 1,021 (84%), and high HDI scores, 1,076 (88.5%). The average age of respondents was 23.44 years (min. 18, max. 55; *SD* = 4.56). A detailed characteristic of the study group is presented in Table 1, and the division of the respondents by HDI and GDP parameters is presented in Tables 2, 3.

**Table 1:** Characteristics of the study group.

	**Number (*n*)**	**Percentage (%)**
**Sex**
Female	904	74.3
Male	312	26.7
**Course of study**
Medicine	860	70.7
Dentistry	78	6.4
Nursing	146	12
Obstetrics	79	6.5
Medical emergency	53	4.4
**GDP income**
High	1,021	84
Upper-middle	86	7.1
Lower-middle	99	8.1
Low	9	0.8
**HDI index**
Very high	1,076	88.5
High	62	5.1
Medium	56	4.6
Low	22	1.8
**Psychiatry course**
Yes	556	45.7
No	660	54.3
**Mental health problems**
Yes	450	37
Yes, in a close relative	388	31.9
No	378	31.1

*GDP, Gross Domestic Product; HDI, Human Development Index*.

**Table 2:** Characteristics of the study group considering the HDI index.

**Variable**		**High**	**Upper-middle**	**Lower-middle**	**Low**	***p***
Sex	Female	837 (77.9%)	38 (61.3%)	25 (44.6%)	3 (13.6%)	**<0.001**
	Male	238 (22.1%)	24 (38.7%)	31 (55.4%)	19 (86.4%)	
Major of medical studies	Medicine	760 (70.7%)	48 (77.4%)	35 (62.5%)	16 (72.7%)	**<0.001**
	Dentistry	69 (6.3%)	7 (11.3%)	0	2 (9.1%)	
	Nursing	120 (11.2%)	7 (11.3%)	19 (33.9%)	0	
	Obstetrics	78 (7.3%)	0	0	1 (4.6%)	
	Medical emergency	48 (4.5%)	0	2 (3.6%)	3 (13.6%)	
Psychiatry course	Yes	499 (46.4%)	31 (50%)	19 (33.9%)	6 (27.3%)	0.071
	No	576 (53.6%)	31 (50%)	37 (66.1%)	16 (72.7%)	
Mental health problems	Yes	434 (40.4%)	11 (17.7%)	2 (3.6%)	3 (13.6%)	**<0.001**
	Yes, in a close relative	346 (32.2%)	16 (25.8%)	17 (30.4%)	8 (36.4%)	
	No	295 (27.4%)	35 (56.5%)	37 (66.1%)	11 (50%)	

*HDI, Human Development Index. Significant effects (<0.05) are marked in bold*.

**Table 3:** Characteristics of the study group considering GDP per capita.

**Variable**		**High**	**Upper-middle**	**Lower-middle**	**Low**	***p***
Sex	Female	805 (78.8%)	54 (62.8%)	42 (42.4%)	2 (22.2%)	**<0.001**
	Male	216 (21.2%)	32 (37.2%)	57 (57.6%)	7 (77.8%)	
Major of medical studies	Medicine	709 (69.4%)	74 (86.1%)	71 (71.8%)	5 (55.6%)	**<0.001**
	Dentistry	66 (69.5%)	8 (9.3%)	3 (3%)	1 (11.1%)	
	Nursing	120 (11.8%)	4 (4.7%)	22 (22.2%)	0	
	Obstetrics	78 (7.6%)	0	0	1 (11.1%)	
	Medical emergency	48 (4.7%)	0	3 (3%)	2 (22.2%)	
Psychiatry course	Yes	475 (46.5%)	39 (45.4%)	37 (37.4%)	4 (44.4%)	0.377
	No	546 (53.5%)	47 (54.7%)	62 (62.6%)	5 (55.6%)	
Mental health problems	Yes	423 (41.4%)	17 (19.8%)	7 (7.1%)	3 (33.3%)	**<0.001**
	Yes, in a close relative	335 (32.8%)	17 (19.8%)	31 (31.3%)	4 (44.5%)	
	No	263 (25.8%)	52 (60.4%)	61 (61.6%)	2 (22.2%)	

*GDP, Gross Domestic Product. Significant effects (<0.05) are marked in bold*.

### Assessment of Students' Attitudes Toward the Psychiatry Domain and Psychiatric Patients

In this study, the mean MICA-2 score obtained by participants was 40.5 ± 9.1 points. Female students obtained significantly lower stigmatization results than male students (39.1 ± 8.2 vs. 44.4 ± 10.2; *p* < 0.001). The lowest extent of stigmatization attitudes was shown by students of the nursing faculties (38.8 ± 7.3), while the highest was by medical emergency students (43.3 ± 10.1).

A statistically significant relationship was observed between the total MICA-2 score and the HDI and GDP per capita scores, where the mean MICA-2 score decreased with increasing HDI (*r* = −0.348; *p* < 0.001) and GDP (*r* = −0.363; *p* < 0.001). Both individuals with a history of mental health problems (38.4 ± 8.5) and those who had relatives suffering from mental health problems (40.2 ± 8.6) showed significantly lower MICA-2 scores than students without such experiences (43.2 ± 9, 5).

There were no group differences regarding having completed a psychiatry course (40.1 ± 9.0 vs. 40.8 ± 9.1; *p* = 0.515). Additionally, regardless of GDP per capita, respondents' previous studies included completed psychiatry classes at similar rates (*p* = 0.337). Detailed results of the analyzed variables are presented in Table 4.

**Table 4:** Results according to specific variables.

**Variable**	**General score**	
	** $\bar{x}$ **	**SD**
**General (*****n*** **=** **1,216)**	40.5	±9.1
**Sex**	
Female (*n =* 904)	39.1	±8.2
Male (*n =* 312)	44.4	±10.2
*P*	**<0.001**
**Major of medical studies**	
Medical (*n =* 860)	39.8	±8.8
Dentistry (*n =* 78)	42.1	±8.2
Nursing (*n =* 146)	43.3	±10.5
Obstetrics (*n =* 79)	38.8	±7.3
Medical emergency (*n =* 53)	43.3	±10.1
*P*	**<0.001**
**GDP income**		
High (*n =* 1,021)	39.3	±8.1
Upper-middle (*n =* 86)	41.3	±9.5
Lower-middle (*n =* 99)	50.3	±10.1
Low (*n =* 9)	55.5	±4.6
*P*	**<0.001**
**HDI index**	
Very high (*n =* 1,076)	39.3	±8.2
High (*n =* 62)	45.3	±11.0
Medium (*n =* 56)	50.9	±9.9
Low (*n =* 22)	54.6	±7.0
*P*	**<0.001**
**Psychiatry course**	
Yes (*n =* 556)	40.1	±9.0
No (*n =* 660)	40.8	±9.1
*P*	0.515
**Mental health problems**	
Yes (*n =* 450)	38.4	±8.5
Yes, in a close relative (*n =* 388)	40.2	±8.6
No (*n =* 378)	43.2	±9.5
*P*	**<0.001**

*GDP, Gross Domestic Product; HDI, Human Development Index*.

There was no significant correlation between the participants' ages and mean MICA-2 scores (*r* = −0.025; *p* = 0.390). Degree of stigma attitude was also analyzed by the MICA-2 single items. Detailed comparisons for single items by GDP per capita and HDI parameters are presented in Supplementary Tables 1, 2, which are provided in the supplementary material for this research.

The results of this study confirm the research hypotheses we have put forward, pointing to the significant dissemination of the phenomenon of stigmatization among students of medical faculties. Moreover, women and inhabitants of cities with higher HDI and GDP/capita show more favorable attitudes.

### ANCOVA Covariance Analysis

The ANCOVA showed group differences in the overall MICA-2 score for age factors, completion of a psychiatry training course and history of a mental disorder, when dividing groups in terms of HDI and GDP per capita parameters. Detailed results of the effects of the confounding factors are presented in Table 5.

**Table 5:** Analysis of covariance (ANCOVA) of potential confounding variables.

**Variable**	**HDI index**		**GDP income**	
	**F**	***p***	**F**	***p***
Sex	0.76	0.512	1.06	0.361
Age	2.00	**0.001**	1.91	**0.008**
Year of studies	1.92	**0.015**	1.28	0.204
Major of medical studies	1.61	0.127	1.64	0.107
Psychiatry course	3.07	**0.011**	5.58	**0.001**
Experience with psychiatric conditions either own or a close relative's	2.29	**0.033**	2.93	**0.006**

*GDP, Gross Domestic Product; HDI, Human Development Index. Significant effects (<0.05) are marked in bold*.

## Discussion

The present study focused on the evaluation of stigma toward the psychiatry domain and patients with psychiatric disorders, as presented by medical university students from 65 countries, taking into account the factors of gender, discipline, whether or not they had finished a training course in psychiatry and individual and/or students' family mental problems. The study also analyzed stigma attitudes internationally by grouping respondents according to the GDP-based income per capita and HDI indices of their home countries (21).

The mean MICA-2 score obtained by the students was 40.05. To the best of the authors' knowledge, this is the first study to include such a diverse population of medical students from medical schools around the world in the context of measuring degree of stigmatization of psychiatry and psychiatric patients using the MICA-2 questionnaire for assessment. So far, available studies have mainly included data on student attitudes from a single academic center or country (12, 16, 17, 22–24).

Amongst the respondents, students from nursing faculties demonstrated the lowest extent of stigmatization, while the highest level of stigma attitudes was observed in medical emergency students. The higher range of hours might be a possible factor in explaining this outcome, as there is a disproportion during these programs in hours spent in direct relation with patients staying in mental health facilities (i.e., prospective nurses usually have more in-person contact with patients, while students of dentistry and medical emergency have rarer contact with patients). The less frequent stigma attitudes might also be attributable to the fact that the overwhelming number of nursing students in the sample (97.6%) were female, and females presented more positive attitudes toward mental illness generally (25, 26). By contrast, medical emergency students are generally males and often have contact only with psychiatric patients with a specific profile. For example, paramedics often deal with patients with psychomotor agitation during emergency medical services, and these patients may also display limitation or abolition of judgment. Moreover, paramedics in some countries are permitted to making use of direct coercion with patients. This might provide medical emergency students with an insufficient and false view of mental illness and contribute to strengthening incorrect stereotypes and increasing their attitudes of stigmatization toward patients (27).

The present results are consistent with earlier reports, in which males revealed a substantially higher degree of stigmatization attitudes than females (12, 15, 16, 28). This might be connected to the fact that males more often perceive mental illness to be a weakness of a personality than women, who tend to associate the presence of mental illness with traumatic events experienced previously in life (29).

The social role theory may offer another explanation for the gender difference. This theory states that females present reduced attitudes of stigmatization toward the psychiatry discipline and mental illness due to their more advanced socialization process in terms of empathy compared to men (30, 31). In addition, other reports have proved that more empathetic attitudes can diminish the influence of negative beliefs about mental illness and encourage healthcare professionals to present more socialized behaviors toward patients, triggering their more positive reactions (26).

An interesting finding is that there was a substantial disproportion between the number of males and females joining the study. Despite the lack of data on the population of female medical students worldwide, one of the most comprehensive reports on this issue seems to be the publication by the Association of American Medical Colleges (AAMC) in 2019, which shows that the number of females going to medical schools in the US is continuously rising. It also shows that the percentage of women is more than 50%, compared to Great Britain and Australia (32–34). For the Polish population of medical students, the percentage of females is as high as 73% (35). There is a noticeable correlation between the HDI index of a given country and the size of the female population attending medical school there. Countries with a high HDI index, such as Norway, are characterized by a substantial bulk of women students (60.6% in Norway) (36), while in countries with a reduced HDI this percentage is lower; for example, in India this percentage is 49% (37). These data are in line with our outcomes, which indicate that reduced HDI countries have less females in medical schools than do nations with a high HDI.

Surprising results in this study were that neither the stage of medical education nor the previous completion of a psychiatry course influenced the final results of the survey. This prompts unfavorable reflection on the quality of psychoeducation in medical universities globally, contrary to the existing literature on the subject that claims that education is intended to make positive adjustments in attitudes toward psychiatry in medical students (38, 39). It therefore appears justified to consider an arrangement by medical universities for appropriate education or counseling to boost the image of psychiatry (or any other specialty) and familiarize students with a possible future career in psychiatry. These measures might change students' attitudes and viewpoints toward various specialties and also would permit a less-biased selection of a specialized career path. Other study reported that students who proclaim to favor the field of expertise in psychiatry have a lower MICA-2 score than students favoring various other specializations of medicine (40).

Interestingly, an innovative Chinese program for mental health care professionals shows results in enhancing knowledge of mental health problems and decreasing negative attitudes toward individuals experiencing mental illness; this program has additionally proved that changes induced by the course can be sustained over time. This program committed a lot more hours to working with patients, health education and rehabilitation training engaging students and patients and long-lasting observation of the patient, as opposed to the traditional training course in psychiatry that focuses primarily on the clinical aspects of illness (41). Therefore, implementing work and community education components in the core psychiatry curriculum may reduce the stigma of mental illness among medical students. In addition, this type of training offers the chance to recognize patients' needs better and monitor the therapy process from a more comprehensive perspective.

Another study on educating American medical students also provides examples of successful education and awareness-raising work to lower stigmatization amongst medical practitioners. This education model was developed to boost communication between physicians, patients with mental diseases and patients' family members on psychiatric topics such as medical diagnosis, treatment process, and stigma. The program additionally consisted of public presentations by family members and representatives of the American National Alliance on Mental Illness (NAMI) organization on topics of communication and ways of minimizing stigma. Furthermore, NAMI speakers emphasized the value of the biological foundations underlying mental illness as a medical paradigm in the search for disease causes and effective treatment instead of stigmatizing or blaming the patients or identifying family dynamics as the etiological basis of mental disease (42).

Other successful approaches to lowering the stigmatizing attitudes of medical students involved seeing documentaries concerning individuals with mental diseases and attending workshops in which people with mental illnesses actively participated (43–45).

The present study found a decrease in stigma attitudes measured by the MICA-2 survey when the GDP per capita and the HDI indices relevant to the respondent's country increased. We believe that this inverse relationship is driven by better access to education in nations with higher HDI values. It is worth mentioning that education parameters are one of the components of the HDI index, defined as a derivative of the average number of education years received by residents aged 25 and older and the expected years of education for children beginning the education process (21).

There are several reports on the beneficial results of diversifying the psychiatry courses in medical schools and using educational training to reduce stigma. For instance, an Australian study on medical students found a decline in the MICA-2 score from 48.2 to 43.5 points after students attended an eight-week course in psychiatry enriched with visits to drug addiction rehab centers and geriatric facilities (3).

One more reason for reduced stigma attitudes in countries with higher GDP and HDI indices is an increased expense on healthcare and psychoeducation. Different social campaigns on anti-discrimination psychoeducation are increasingly showing up in the general public space in industrialized nations, primarily focussed on depression (46, 47). For example, the “Open the Doors” initiative of the World Psychiatric Association aimed at reducing the stigmatization of mental disorders experienced by individuals had a measurable impact on the application of anti-discrimination programs in youth communities; the effectiveness of the “Open the Doors” program was recognizable in several countries (48). On the other hand, in 2013, a study by Friedrich et al. showed that anti-discrimination training among British medical students had only a short-term impact on MICA-2 outcomes as confirmed by the follow-up after six months (49).

In this present study, both respondents who had histories of mental health issues and those who had relatives suffering from mental health problems showed substantially lower MICA-2 scores than those who did not. These outcomes are consistent with previous studies indicating that specific experience of mental disorders lowers the stigmatization of mental health problems (12, 16, 17, 22–24).

The authors recognize the limitations of this study. The first limitation is that the data collection was based on social media and online circulation of surveys on the internet. Nonetheless, due to the present circumstance of the pandemic, online surveys are a safe and also conveniently accessible research method that permits obtaining a large number of recipients. They also allow accessibility across the world population of medical university students. On the other hand, it is not feasible to confirm the accuracy of the data, including personal information, the real number of recipients that received information about the study, and the number of started but unfinished surveys. Another limitation was the fact that that the Mental Illness: Clinicians' Attitudes (MICA-2) scale didn't go through the process of cross-cultural validation.

This study was also time limited, which did not allow determination of whether the opportunity to interact with mentally ill individuals during the study contributed to stigmatizing views among students or whether these views existed prior to the study. We are likewise unable to determine to what extent stigma attitudes and beliefs might have altered during a training course in psychiatry. However, it is believed that medical students' attitudes toward mentally ill individuals often decrease over time (39, 50, 51). Following the same cohort throughout their period of study and assessing their views each year would be useful in developing appropriate teaching resources targeted at each age group according to their needs and demands. Further considerable limitations of the present study involve the representativeness of the investigated group, notably the predominance of females in the sample, the large proportion of students from countries with high HDI and GDP per capita indices and the digital exclusion of prospective respondents from lower wealthy countries; all of these factors might have affected the final results of the analysis.

The last couple of years have brought massive changes in the mental health care system, including the closures of old psychiatric centers, the expansion of the community care sector and the number of non-pharmacological treatments. A variety of activities have also been undertaken to de-stigmatize the field of psychiatry and psychiatric patients. As a result, progress has been made in understanding the dimensions of mental disorder stigma and the recognition of processes through which public stereotypes convert into discriminatory behaviors. Nevertheless, the activities aimed at increasing public awareness of the role of psychiatry are still not enough and, indeed, in some countries, entirely missing.

The research on student groups and young medical professionals is of great significance in the context of changes in the perception of psychiatry in society as their attitudes and interactions with patients and their family members contribute to lowering discrimination and prejudices and weakening the self-stigmatization of patients. Nevertheless, it appears that some damaging stereotypes still remain among medical students.

These observations are particularly essential in the context of recent research on the global crisis of mental health resulting from the COVID-19 pandemic. It has been revealed that the COVID-19 pandemic has substantially affected social functioning from its beginning. This has resulted in an increase in anxiety and depression symptoms in Poland compared to the pre-pandemic period (52–56).

Undoubtedly, the psychiatry curriculum at universities should focus on understanding the problems faced by patients and the mental health care system. It is possible that prior to the very first clinical contact with psychiatric patients, students need to be familiarized with principles of good practice for contact and care of patients, with particular attention paid to the specifics of mental disorders and related symptoms presented by patients.

## Conclusions

The results of this study indicate the widespread occurrence of stigmatization of both psychiatry and psychiatric patients by medical students in several countries. Women students and individuals who have themselves experienced mental illnesses show more favorable attitudes, as do the inhabitants of countries with high HDI and GDP per capita values. There is a need to further deepen the research into stigmatization, both among the general population and medical personnel, and implement and maintain appropriate measures to reduce stigma toward mental illness and psychiatry.

## Data Availability Statement

The raw data supporting the conclusions of this article will be made available by the authors, without undue reservation.

## Ethics Statement

The studies involving human participants were reviewed and approved by Bioethics Committee of the Wroclaw Medical University, Poland (approval no: KB-178/2021). Written informed consent for participation was not required for this study in accordance with the national legislation and the institutional requirements.

## Author Contributions

KK, BB, and PP: conceptualization. MB: data curation. MB and KK: formal analysis. MB, MM, BB, and PP: investigation. MB, MM, KK, BB, and PP: methodology and writing—review and editing. MB and PP: supervision. MM: validation. BB and PP: visualization. MB, MM, KK, and BB: writing—original draft. All authors contributed to the article and approved the submitted version.

## Conflict of Interest

The authors declare that the research was conducted in the absence of any commercial or financial relationships that could be construed as a potential conflict of interest.

## Publisher's Note

All claims expressed in this article are solely those of the authors and do not necessarily represent those of their affiliated organizations, or those of the publisher, the editors and the reviewers. Any product that may be evaluated in this article, or claim that may be made by its manufacturer, is not guaranteed or endorsed by the publisher.
