# Supplementary material for: Stigma Levels Toward Psychiatric Patients Among Medical Students—A Worldwide Online Survey Across 65 Countries
Source: Front Psychiatry. 2021 Dec 13;12:798909. doi: 10.3389/fpsyt.2021.798909 (PMC8710677; doi:10.3389/fpsyt.2021.798909)
Supplement: Supplementary file 1 [file Data_Sheet_1.docx]

Supplementary Material

**Supplementary Files**

**Table S1.** Distribution of responses to the MICA-2 questionnaire concerning the GDP per capita.

| **Item** | **High** | | **Upper-middle** | | **Lower-middle** | | **Low** | | ***p*** |
| --- | --- | --- | --- | --- | --- | --- | --- | --- | --- |
|  | Agree * | Dis-agree** | Agree * | Dis-agree** | Agree * | Dis-agree** | Agree * | Dis-agree** |  |
| 1. I just learn about psychiatry because it is in the exam and would not bother reading additional material on it. | 253 (24.8%) | 768  (75.2%) | 22  (25.6%) | 64  (74.4% | 54  (54.5%) | 45  (45.5% | 7  (77.8%) | 2  (22.2%) | **<0.001** |
| 2. People with a severe mental illness can never recover enough to have a good quality of life. | 207  (20.3%) | 814  (86.2%) | 21  (24.4% | 65  (75.6% | 38  (38.4%) | 61  (61.6%) | 5  (55.6%) | 4  (44.4%) | **<0.001** |
| 3. Psychiatry is just as scientific as other fields of medicine. | 920  (90.1%) | 101  (9.9%) | 77  (89.5%) | 9  (10.5%) | 81  (81.8%) | 18  (18.2%) | 8  (88.9%) | 1  (11.1%) | 0.08 |
| 4. If I had a mental illness, I would never admit this to any of my friends because I would fear being treated differently. | 315  (30.9%) | 706  (69.1%) | 32  (37.2%) | 54  (62.8%) | 52  (52.5%) | 47  (47.5%) | 6  (66.7%) | 3  (33.3%) | **<0.001** |
| 5. People with a severe mental illness are dangerous more often than not. | 374  (36.6%) | 647  (63.4%) | 30  (34.9%) | 56  (65.1%) | 73  (73.7%) | 26  (26.3%) | 8  (88.9%) | 1  (11.1%) | **<0.001** |
| 6. Psychiatrists know more about the lives of people treated for a mental illness than do family members or friends. | 712  (69.7%) | 309  (30.3%) | 64  (74.4%) | 22  (25.6%) | 82  (82.8%) | 17  (17.2%) | 7  (77.78%) | 2  (22.2%) | 0.04 |
| 7. If I had a mental illness, I would never admit this to my colleagues for fear of being treated differently. | 472  (46.2%) | 549  (53.7%) | 47  (54.6%) | 39  (45.4%) | 55  (55.6%) | 44  (44.4%) | 4  (44.4%) | 5  (55.6%) | 0.17 |
| 8. Being a psychiatrist is not like being a real doctor. | 44  (4.3%) | 977  (95.7%) | 8  (9.3%) | 78  (90.7%) | 31  (34.9%) | 68  (68.7%) | 6  (66.7%) | 3  (33.3%) | **<0.001** |
| 9. If a consultant psychiatrist instructed me to treat people with a mental illness in a disrespectful manner. I would not follow their instructions. | 885  (86.7%) | 136  (13.3%) | 69  (80.2%) | 17  (19.8%) | 80  (80.8%) | 19  (19.2%) | 8  (88.9%) | 1  (11.1%) | 0.17 |
| 10. I feel as comfortable talking to a person with a mental illness as to a person with a physical illness. | 690  (67.6%) | 331  (32.4%) | 67  (77.9%) | 19  (22.1%) | 70  (70.7%) | 29  (30%) | 5  (55.6%) | 4  (44.4%) | 0.18 |
| 11. It is important that any doctor supporting a person with a mental illness also assesses their physical health. | 1005  (98.4%) | 16  (1.6%) | 84  (97.7%) | 2  (2.3%) | 91  (91.9%) | 8  (8%) | 9  (100%) | 0  (0%) | **<0.001** |
| 12. The public does not need to be protected from people with a severe mental illness. | 506  (49.6%) | 515  (50.4%) | 35  (40.7%) | 51  (59.3%) | 29  (30%) | 70  (70.7%) | 6  (66.7%) | 3  (0.5%) | **<0.001** |
| 13. If a person with a mental illness complained of physical symptoms (such as chest pain). I would attribute it to their mental illness. | 240  (23.5%) | 781  (76.5%) | 24  (27.9%) | 62  (72.1%) | 27  (27.3%) | 72  (72.7%) | 4  (44.4%) | 5  (55.6%) | 0.33 |
| 14. General practitioners should not be expected to complete a thorough assessment for people with psychiatric symptoms because they can be referred to a psychiatrist. | 323  (31.6%) | 698  (68.4%) | 37  (43%) | 49  (60%) | 60  (60.6%) | 39  (39.4%) | 8  (88.9%) | 1  (11.1%) | **<0.001** |
| 15. I would use the terms ‘crazy’, ‘nutter’, ‘mad’, etc. to describe people with a mental illness who I have seen in my work | 81  (7.9%) | 940  (92.1%) | 7  (8.1%) | 79  (91.9%) | 30  (30.3%) | 69  (69.7%) | 6  (66.7%) | 3  (33.3%) | **<0.001** |
| 16. If a colleague told me they had a mental illness, I would still want to work with them. | 991  (97%) | 30  (2.9%) | 85  (98.8%) | 1  (1.2%) | 81  (81.8%) | 18  (18.2%) | 8  (88.9%) | 1  (11.1%) | **<0.001** |
| * I strongly agree / I agree / I rather agree  ** I strongly disagree / I disagree/ I rather disagree | | | | | | | | | |

*Abbreviations:* GDP, Gross Domestic Product; MICA-2, Mental Illness: Clinicians’ Attitudes.

**Table S2.** Distribution of responses to the MICA-2 questionnaire concerning the HDI index.

| **Item** | **Very high** | | **High** | | **Medium** | | **Low** | | ***p*** |
| --- | --- | --- | --- | --- | --- | --- | --- | --- | --- |
|  | Agree * | Dis-agree** | Agree * | Dis-agree** | Agree * | Dis-agree** | Agree * | Dis-agree** |  |
| 1. I just learn about psychiatry because it is in the exam and would not bother reading additional material on it. | 267 (24.8%) | 808 (75.2%) | 26 (41.9%) | 36 (58.1%) | 31 (55.4%) | 25 (44.6%) | 12 (54.6%) | 10 (45.4%) | **<0.001** |
| 2. People with a severe mental illness can never recover enough to have a good quality of life. | 218 (20.3%) | 857 (79.7%) | 20 (32.3%) | 42 (67.7%) | 23 (41.1%) | 33 (58.9%) | 10 (45.4%) | 12 (54.6%) | **<0.001** |
| 3. Psychiatry is just as scientific as other fields of medicine. | 970 (90.2%) | 105 (9.8%) | 50 (80.7%) | 12 (19.3%) | 47 (83.9%) | 9 (16.1%) | 19 (86.4%) | 3 (13.6%) | 0.082 |
| 4. If I had a mental illness, I would never admit this to any of my friends because I would fear being treated differently. | 335 (31.2%) | 740 (68.8%) | 27 (43.6%) | 35 (56.4%) | 30 (53.6%) | 26 (46.4%) | 13 (59.1%) | 9 (40.9%) | **<0.001** |
| 5. People with a severe mental illness are dangerous more often than not. | 388 (36.1%) | 687 (63.9%) | 38 (61.3%) | 24 (38.7%) | 42 (75%) | 14  (25%) | 17 (77.3%) | 5 (22.7%) | **<0.001** |
| 6. Psychiatrists know more about the lives of people treated for a mental illness than do family members or friends. | 750 (69.8%) | 325 (30.2%) | 48 (77.4%) | 14 (22.6%) | 47 (83.9%) | 9 (16.1%) | 20 (90.9%) | 2 (9.1%) | **0.006** |
| 7. If I had a mental illness, I would never admit this to my colleagues for fear of being treated differently. | 502 (46.7%) | 573 (53.3%) | 33 (53.2%) | 29 (46.8%) | 32 (57.1%) | 24 (42.9%) | 11 (50%) | 11 (50%) | 0.357 |
| 8. Being a psychiatrist is not like being a real doctor. | 48 (4.5%) | 1027 (95.5%) | 10 (16.1%) | 52 (83.9%) | 20 (35.7%) | 36 (64.3%) | 11 (50%) | 11 (50%) | **<0.001** |
| 9. If a consultant psychiatrist instructed me to treat people with a mental illness in a disrespectful manner, I would not follow their instructions. | 933 (86.8%) | 142 (13.2%) | 45 (72.6%) | 17 (27.4%) | 46 (82.1%) | 10 (17.9%) | 18 (81.8%) | 4 (18.2%) | **0.028** |
| 10. I feel as comfortable talking to a person with a mental illness as to a person with a physical illness. | 733 (68.2%) | 342 (31.8%) | 43 (69.4%) | 19 (30.6%) | 42 (75%) | 14  (25%) | 14 (63.6%) | 8 (36.4%) | 0.694 |
| 11. It is important that any doctor supporting a person with a mental illness also assesses their physical health. | 1057 (98.3%) | 18 (1.7%) | 61 (98.4%) | 1 (1.6%) | 50 (89.3%) | 6 (10.71%) | 21 (95.5%) | 1 (4.5%) | **0.007** |
| 12. The public does not need to be protected from people with a severe mental illness. | 526 (48.9%) | 549 (51.1%) | 23 (37.1%) | 39 (62.9%) | 15 (26.8%) | 41 (73.2%) | 12 (54.5%) | 10 (45.5%) | **0.002** |
| 13. If a person with a mental illness complained of physical symptoms (such as chest pain). I would attribute it to their mental illness. | 255 (23.7%) | 820 (76.3%) | 15 (24.2%) | 47 (75.8%) | 14 (25%) | 42  (75%) | 11 (50%) | 11 (50%) | 0.073 |
| 14. General practitioners should not be expected to complete a thorough assessment for people with psychiatric symptoms because they can be referred to a psychiatrist. | 348 (32.4%) | 727 (67.6%) | 28 (45.2%) | 34 (54.8%) | 34 (60.7%) | 22 (39.3%) | 18 (81.8%) | 4 (18.2%) | **<0.001** |
| 15. I would use the terms ‘crazy’, ‘nutter’, ‘mad’, etc. to describe people with a mental illness who I have seen in my work | 83 (7.7%) | 992 (92.3%) | 10 (16.1%) | 52 (83.9%) | 20 (35.7%) | 36 (64.3%) | 11 (50%) | 11 (50%) | **<0.001** |
| 16. If a colleague told me they had a mental illness, I would still want to work with them. | 1044 (97.1%) | 31 (2.9%) | 59 (95.2%) | 3 (4.9%) | 47 (83.9%) | 9 (16.1%) | 15 (68.2%) | 7 (31.8%) | **<0.001** |
| * I strongly agree / I agree / I rather agree  ** I strongly disagree / I disagree/ I rather disagree | | | | | | | | | |

*Abbreviations:* HDI, Human Development Index; MICA-2, Mental Illness: Clinicians’ Attitudes.
